# Galectin 3 rs4644 gene polymorphism is associated with metabolic traits in Serbian adolescent population

**DOI:** 10.5937/jomb0-47180

**Published:** 2024-06-15

**Authors:** Vanja Vidović, Ivana Novaković, Tatjana Damnjanović, Zana Radić-Savić, Stojko Vidović, Ranko Škrbić, Nela Maksimović

**Affiliations:** 1 University of Banja Luka, Faculty of Medicine, Department of Human Genetics, Banja Luka, Bosnia and Herzegovina; 2 University of Banja Luka, Faculty of Medicine, Center for Biomedical Research, Laboratory for Molecular Biology and Genetics, Banja Luka, Bosnia and Herzegovina; 3 University of Belgrade, Faculty of Medicine, Institute of Human Genetics, Belgrade; 4 University of Banja Luka, Faculty of Medicine, Department of Medical Biochemistry, Banja Luka, Bosnia and Herzegovina; 5 University of Banja Luka, Faculty of Medicine, Department of Pharmacology, Toxicology and Clinical Pharmacology, Banja Luka, Bosnia and Herzegovina

**Keywords:** BMI, LGALS3, rs4644, lipid profile, ITM, LGALS3, rs4644, lipidni status

## Abstract

**Background:**

Among many genes which have been analyzed to understand obesity and related metabolic traits among children and adolescents, not many studies are conducted on LGALS3 gene, especially in population of children. A positive correlation of circulating galectin 3 serum levels with impaired blood glucose, high blood pressure and higher values of serum lipids and was found in general population. The aim was to investigate possible association of rs4644 with body mass index, glycaemia, and lipid profile in Serbian adolescents.

**Methods:**

The study included 72 boys and 79 girls, 14-15 years of age. Among boys 51 (67.1%) had normal values of BMI, 11 (14.5%) were overweight, and 14 (18.4%) were obese. Among girls, 53 (63.9%) had normal BMI, 16 (19.3%) were overweight, and 14 (16.9%) were obese. Diabetes type 1 or 2, genetic syndromes, generalized inflammation, cardiovascular and malignant diseases were criteria for exclusion. Genotyping was performed by Real time PCR.

## Abstract (sr)

**Uvod:**

Među brojnim genima koji se analiziraju kako bi se razumela genetička osova gojaznosti i metaboličkih parametara, vrlo je malo studija koje su istraživale ulogu LGALS3 gena, posebno u populaciji dece i adolescenata. U opštoj populaciji utvrđena je pozitivna korelacija nivoa cirkulišućeg galaktina sa poremećenim nivoom glukoze u krvi, visokim krvnim pritiskom i višim vrednostima lipida u serumu. Cilj ove studije je bio da se ispita moguća povezanost rs4644 polimorfizma sa indeksom telesne mase, glikemijom i lipidnim statusom kod adolescenata u Srbiji.

**Metode:**

Studija je obuhvatila 72 dečaka i 79 devojčica uzrasta 15 godina. U grupi dečaka 51 (67,1%) je imalo normalan ITM, 11 (14,5%) su bili preuhranjeni, dok je 14 (18,4%) bilo gojazno. U grupi devojčica, 53 (63,9%) je imalo normalan ITM, 16 (19,3%) su bile preuhranjene, dok je 14 (16,9%) bilo gojazno. Diabetes tip 1 ili 2, genetički sindromi, sistemska zapaljenja, kardiovaskularne i maligne bolesti su bili kriterijumi za isključivanje iz studije. Genotipizacija je vršena pomoću Real time PCR-a.

**Rezultati:**

Devojčice nosioci CC genotipa su imale statistički više vrednosti ITM i triglicerda u odnosu na devojčice nosioce CA+AA genotipova, p=0,041 i p=0,045, respektivno. Veća učestalost gojaznosti je utvrđena u grupi devojčica koje su bile nosioci CC genotipa, p=0,049. Nije uočena statistički značajna povezanost između ostalih analiziranih parametara ni u jednoj ispitivanoj grupi.

**Zaključak:**

Naše istraživanje ukazuje na povezanost između CC genotipa rs4644 polimorfizma sa gojaznošću i višim nivoom srednjih vrednosti triglicerida u grupi devojčica.

## Introduction

Children and adolescent obesity has become a worldwide problem, with high incidence not only in the western world, but also in developing countries. Problem of obesity is not only excessed body weight, but also risk factors for developing a metabolic syndrome (MetS) [1]. Metabolic syndrome includes several factors such as glucose intolerance hypertension, insulin resistance, dyslipidemia, and diabetes mellitus type 2 [2]. Since for obesity is characterized hypertrophy of white adipose tissue, macrophage together with adipocyte are secreting pro-inflammatory cytokines and chemokines, thus exceeded body weight might also have impact on inflammatory status, which is in correlation with decreased glucose tolerance and insulin sensitivity [3]. Among many genes which have been analyzed in order to understand obesity and related metabolic traits among children and adolescent, not many studies are conducted on *LGALS3* gene, particularly in population of children. However, Galectin 3 (Gal-3) is a protein encoded by *LGALS3* gene located on the chromosome 14 [4]. This protein belongs to the family of β-galactoside-binding lectins which counts fourteen more members. Gal-3 is involved in many metabolic processes including basic physiological processes such as cell proliferation, adhesion and programed cell death. Also, in humans, Gal-3 is expressed in various tissues such as epithelial cells, mast cells, immune cells including monocytes, macrophages, dendritic cell, etc [5]. Previous researchers have found a positive correlation of circulating galectin 3 serum levels in general population with higher risk of cardiovascular, renal diseases, high blood pressure as well as with higher values of serum lipids and obesity [6]. Also, galactines promote preadipocytes maturation into the lipid-loaded adipocyte, which might contribute to central obesity [7]. Moreover, deletion of *LGALS3* gene in mice have shown defectiveness in adipose tissue maturation, which was accompanied by increased body inflammation, higher glucose level and impaired insulin sensitivity [8]. Within the *LGALS3* gene, common single nucleotide polymorphisms (SNP) have been described as rs4644 C > A, which leads to the substitution of histidine to proline at residue 64 [9]. This SNP have been linked with higher serum levels of galactin 3 protein [9] [10]. The aim of this research was to investigate possible association of rs4644 with BMI (body mass index), glycaemia, and lipid profile in Serbian adolescents.

## Materials and methods

### Research design

Participants enrolled in the study were children 14 to 15 years of age, of both genders who were randomly selected from Yugoslav Study of the Precursors of Atherosclerosis in School Children (YUSAD). Among selected children (*n*=151), 72 were boys and 79 were girls. Anthropometric data including age, gender, height, weight as well as biochemical parameters such as fasting blood glucose (FBG), total cholesterol (TC), triglycerides (TG), high density lipoprotein cholesterol (HDL-C), and low density lipoprotein cholesterol (LDL-C) values were recorded during annual pediatric examinations at 11 primary health care centers from all parts of Serbia. Children’s BMI was classified in three different groups, according to the previously published charts for Serbian adolescents (normal weight, overweight above 85^th^ percentile, and obese above 95^th^ percentile [11]. Ethics Committee of Faculty of Medicine, University of Belgrade, Serbia approved study design and study protocol, (approval number 29/X-9 from 12.10.2017.). For all children included in the study, parents or guardians signed informed consent form. Selected criteria for exclusion from the study were cardiovascular, malignant, genetic diseases as well as general inflammation, both types of *diabetes mellitus*, cerebral palsy and chronic immobility.

### Biochemical and molecular–genetic analysis

Prior to the blood sample collection, all participant fasted for 12 hours. Fasting blood glucose, TC, TG, HDL-C values were measured as described previously, and LDL-C values were calculated by Friedwald’s equation [11]. Molecular-genetic analysis were performed at the Institute of Human Genetics, Faculty of Medicine, University of Belgrade, Serbia. Prior to genotyping, the total genomic DNA was isolated by salting out method [12]. Genotyping was performed by Real time PCR using available commercial assay (C___7593635_1), according to the manufacture's protocol (Applied Biosystems, Foster City, CA, USA). The results were analyzed using Applied Biosystems 7500 software v2.0.6 (Applied Biosystems, Foster City, CA). Data were analyzed by statistical SPSS (version 17) software.

### Statistical analysis

Quantitative variables were expressed as mean ± standard deviation. Depending on the variable distribution, Student’s t-test or the Mann-Whitney test was used to assess the association of BMI, fasting blood glucose and lipid parameters with selected genotype.

## Results

The enrolled participant in the study were 14– 15 years old children of both genders. The total number of children was (*n* = 151), of which 72 (47.6%) were boys, and 79 (52.3%) were girls. Among boys 51 (67.1%) had normal values of BMI, 11 (14.5%) were overweight, and 14 (18.4%) were obese. Among girls, 53 (63.9%) had normal BMI, 16 (19.3%) were overweight, and 14 (16.9%) were obese. Mean values of analyzed parameters are presented in Table 1. Statistical significance was observed among mean values of total cholesterol and high density lipoprotein cholesterol, where girls had higher mean values compared to the group of boys ((p=0.014, p=0.009, respectively). No statistical significance was reached among other analyzed parameters. Frequencies of genotypes and alleles are presented in Table 2.

**Table 1:** Mean values and range of analyzed parameters depending on gender. BMI: body mass index; FBG: fasting blood glucose; TG: triglycerides; TC: statistically significant total cholesterol; HDL-C: high-density lipoprotein cholesterol; LDL-C: low-density lipoprotein cholesterol.

Parameters	Total (n = 151) (range)	Boys (n = 72)	Girls (n = 79)	p Value
BMI (kg/m^2^)	22.36±15.32 (13.8–43.72)	22.00±4.50	22.69±4.71	0.330
FBG (mmol/L)	4.68±0.55 (3.20–6.30)	4.72±0.54	4.64±0.57	0.345
TG (mmol/L)	1.01±0.62 (0.28–5.40)	1.02±0.71	1.01±0.53	0.421
TC (mmol/L)	4.53±0.92 (0.90–6.70)	4.33±0.87	4.71±0.93	0.014
HDL-C (mmol/L)	1.41±0.42 (0.28–4.00)	1.34±0.46	1.47±0.38	0.009
LDL-C (mmol/L)	2.67±0.88 (0.74–5.28)	2.61±0.88	2.74±0.88	0.320

**Table 2:** Frequencies of LGALS3 genotypes and alleles.

*LGALS3* rs4644 Genotypes	Frequency <br>n (%)	Alleles	Frequency (%)
CC	85 (56.3)	C	73
CA	50 (33.1)	–	–
AA	16 (10.6)	A	27

For further analysis we grouped CA and AA genotypes. In line with that, statistically significant results were obtained among group of girls carriers CC genotype, where they had higher BMI in comparison to the girls carriers of CA+AA genotypes (p=0.041) . Also, in the same analyzed group, girls carriers of CC genotype had statistically higher mean values of TG compared to the girls carriers of CA+AA genotypes (p=0.045). No other statistically significance was obtained among other analyzed parameters in neither groups, regarding rs4644 genotype, which is presented in Table 3.

**Table 3:** Mean values of analyzed parameters by LGALS3 rs4644 genotype (mean±SD). BMI: body mass index; FBG: fasting blood glucose; TG: triglycerides; TC: statistically significant total cholesterol; HDL-C: high-density

Parameters	Genotype	Total	*p* Value	Boys	p Value	Girls	*p* Value
BMI (kg/m^2^)	CCCA+AA	22.61±4.71 <br>22.05±4.48	0.461	21.56±3.98 <br>22.71±5.20	0.374	23.73±5.21 <br>21.56±3.86	0.041
FBG (mmol/L)	CCCA+AA	4.63±0.57 <br>4.74±0.53	0.143	4.64±0.60 <br>4.84±0.39	0.189	4.62±0.55 <br>4.67±0.59	0.731
TG (mmol/L)	CCCA+AA	1.03±0.59 <br>0.99±0.66	0.626	0.94±0.52 <br>1.13±0.93	0.361	1.12±0.64 <br>0.88±0.33	0.045
TC (mmol/L)	CCCA+AA	4.55±0.82 <br>4.50±1.04	0.845	4.36±0.76 <br>4.27±1.04	0.651	4.75±0.84 <br>4.67±1.03	0.735
HDL-C (mmol/L)	CCCA+AA	1.38±0.36 <br>1.44±0.49	0.691	1.31±0.36 <br>1.39±0.59	0.995	1.45±0.35 <br>1.49±0.35	0.598
LDL-C (mmol/L)	CCCA+AA	2.69±0.84 <br>2.66±0.93	0.835	2.65±0.90 <br>2.55±0.85	0.643	2.73±0.78 <br>2.74±0.99	0.856

## Discussion

In the recent years many studies are conducted on human and animal models, which have found the positive correlation of higher circulating levels of Gal- 3 protein and impaired glucose metabolism, insulin sensitivity, obesity, hypercholesterolemia, and inflammation factors [13]. The level of its expression varies through the lifespan, with the highest levels of circulating Gal-3 during embryogenesis and development. Higher serum values of circulating protein were found in obese and diabetic patients in comparison to the nondiabetic patient [14] [15] [16] [17] [18]. Also, animal models are suggesting that the elevated levels of galectin-3 found in the altered metabolic processes such as lipid metabolism, obesity, diabetes and metabolic syndrome might be acting at the level of pancreatic islets and adipose tissues [4] [13]. The study of Liao et al. [19] conducted in a group of patient with coronary artery disease have found the association of rs4644 CC genotype with higher galectin-3 levels, followed by CA and AA genotype. The results of our research regarding BMI and its associations with rs4644 genotype have showed a statistical significance among girls carriers of CC genotype with higher BMI in comparison to the girls who were carriers of CA+AA genotype (p=0.041). The higher frequency of obese was found among group of girls who were carriers of CC genotype (p=0.049). No association was found among the group of boys regarding BMI and rs4644 genotype. Moreover, girl carriers of CC genotype also had statistically higher mean values of TG in comparison to the girls carriers of CA or AA genotype (p=0.045). No association was found among group of boys in neither analyzed parameters. Since, studies have found two common SNPs (rs4644 and rs4652) are affecting the protein properties of the *LGALS3* gene product, leading to the higher expression of Gal-3 protein [5] [6] [7], research of Florido et al. [20] have shown that higher BMI was in direct correlation with elevated galectin-3 levels. Also, patients with severe obesity and elevated galectin-3 had >4-fold higher risk of developing heart failure. One of the studies conducted by Zhen et al [7], reported elevated expression of Galectin-3BP and positive correlation with obesity and MetS in 932 Chinese adolescents. On the other side Besma et al. [21] obtained no statistically significant results regarding BMI among patients with type 2 *diabetes mellitus* regarding rs4644 genotype. One of the possible explanation of these results are differences in gonadal hormonal status. Namely, in the puberty increased secretion of progesterone, estrogen and androgen hormones in female compared to adolescent boys, which might lead to increased BMI and visceral adiposity [22]. Also, in the period of adolescence, boys tend to have more physical activities than girls, which might be one of the reason for difference in BMI [23]. On the other side impact of increased secretion of testosterone in adolescent boys, leads to the higher lipase activity in the liver, which causes lowering of the lipid parameters as well as lower BMI [24]. However, many of these studies measured the circulating level of protein in particular populations, but association of rs4644 genotype was not addressed. Also, available data indicated that these studies were conducted in the population of adult patients, but not many data are available in the population of children and adolescents. However, the importance of association studies in children and adolescents refereeing to rs4644 polymorphism is reflecting on the comorbidities accompanying elevated BMI and lipid parameters primarily with cardiovascular and metabolic diseases since many studies showed association of this polymorphic variant with insulin resistance, diabetes mellitus type 2 and cardiovascular events [9] [25] [26]. Thus, the importance of monitoring these biochemical parameters in adolescents might contribute to the lowering incidence of metabolic syndrome and other diseases associated with obesity-related parameters.

## Conclusion

In this research we have found a statistically significant correlation between CC genotype of rs4644 polymorphism with higher BMI and mean values of triglyceride levels. However, one of the limitations of the study was relatively small sample size, and lack of non-genetic information such as eating habits, or level of physical activity. For further analysis and better understanding of children and adolescent obesity and other metabolic parameters, non-genetic factors and other polymeric variants among *LGLAS3* gene, as well as other genetic variants should be taken into consideration.

## Dodatak

### Conflict of interest

None.

### Acknowledgements

The research was granted by the Serbian Ministry of Education, Science and Technological development (Grant 175091).

### Authorship Contributions

All authors contributed equally to the concept, processing, analysis and writing of this paper.

### Conflict of interest statement

All the authors declare that they have no conflict of interest in this work.

### List of abbreviations

Gal3 – Galectin 3,<br>MetS – metabolic syndrome, <br>BMI – body mass index,<br>YUSAD – Yugoslav Study of the Precursors of Atherosclerosis in School Children,<br>FBG – fasting blood glucose,<br>TC – total cholesterol,<br>TG – triglycerides, <br>HDL-C – high density lipoprotein cholesterol,<br>LDL-C – low density lipoprotein cholesterol,<br>SNP – single nucleotide polymorphism,<br>PCR – polymerase chain reaction
